# New Evidence on Carbetocin: Another Arrow in Our Quiver

**DOI:** 10.9745/GHSP-D-18-00336

**Published:** 2018-10-03

**Authors:** Steve Hodgins

**Affiliations:** a Editor-in-Chief, Global Health: Science and Practice Journal, and Associate Professor, School of Public Health, University of Alberta, Edmonton, Alberta, Canada.

## Abstract

Carbetocin is more heat stable than oxytocin with at least equivalent efficacy for preventing postpartum hemorrhage. It will certainly be helpful if the supplier can make it available in low-income country settings at a price comparable to oxytocin. But even so, programs will still need oxytocin and other uterotonic medications.

On August 23, 2018, the *New England Journal of Medicine* published the results of the large, multi-country, World Health Organization (WHO) CHAMPION trial,^1^ a non-inferiority study testing carbetocin against synthetic oxytocin for prevention of postpartum hemorrhage. Publication of this trial created some buzz in the international media, with reports in BBC and the *New York Times*, including quotes from WHO officials claiming that wider use of carbetocin could “revolutionize our ability to keep mothers … alive.”^2^ That may be claiming too much.

Uterotonics have a critically important role in obstetrics, notably for labor induction and augmentation and for prevention and treatment of postpartum hemorrhage. Whatever the indication, in using these drugs clinicians seek to optimize for *efficacy* and *safety*, taking into account—among other considerations—characteristics of the specific drugs, dosage, route of administration, clinical indications, and patient characteristics.

Synthetic oxytocin has been widely used in obstetrical practice since the 1960s. Carbetocin—more recently introduced—is an oxytocin analog, acting on oxytocin receptors in the myometrium, but in some important respects it differs from synthetic oxytocin: its half-life in circulation is considerably longer and it is more heat stable.^3^

## CLINICAL EFFICACY

Heat-related degradation of oxytocin is well-documented,^4^ and studies in both Africa^5^ and South Asia^6^ have found a significant proportion of oxytocin sold at retail level falling outside of manufacturer specifications. The greater heat stability of carbetocin means there is more certainty about the actual delivered dose than there is for oxytocin. But this doesn't necessarily translate into any significant difference in *clinical efficacy*. In the new study, the authors claim oxytocin has “unsatisfactory real-world efficacy as a result of sensitivity to heat.”^1^ However, the evidence they cite consists only of assays of the amount of active pharmaceutical ingredient in the vials sampled,^7^ *not* effects on patient outcomes.

A recently published trial^8^ provides good evidence that route of administration matters; in a double-blinded, head-to-head comparison of oxytocin 10 IU administered intravenously versus intramuscularly, the investigators found significant differences in clinically important endpoints, with better results for intravenous administration than for intramuscular for blood loss ≥1000 cc (adjusted odds ratio [aOR], 0.54; 95% confidence interval [CI], 0.32 to 0.91) and need for transfusion (aoR, 0.31; 95% CI, 0.13 to 0.70). So route of administration matters. But no similarly unequivocal evidence is available for a difference in clinical efficacy between 5 IU and 10 IU. A Cochrane review by Westhoff^9^ found 5 trials comparing either 5 IU or 10 IU to placebo. In a pooled comparison, with blood loss ≥500 cc as the endpoint (Analysis 2.4), pooled effect sizes were similar for 5 IU (relative risk [RR], 0.42; 95% CI, 0.17 to 1.01) and 10 IU (RR, 0.47; 95% CI, 0.38 to 0.59). The Table looks a little more closely at these trials.

**TABLE:** Summary of Current Evidence on 5 IU or 10 IU Oxytocin Compared With Placebo

Study	N	Route of Administration	Dose (IU)	RR for Blood Loss ≥500 cc (95% CI)	Other Clinically Important Endpoints: RR (95% CI)	Methodologic Issues
De Groot (1996)^10^	221	IM	5	0.83 (0.57, 1.22)	Blood loss ≥1000 cc: 0.80 (0.34, 1.87) Therapeutic uterotonic needed: 0.99 (0.55, 1.78)	Provisions for blinding not specified
Poeschmann (1991)^11^	52	IM	5	0.60 (0.27, 1.33)	Blood loss ≥1000 cc: 0.57 (0.10, 3.14) Therapeutic uterotonic needed: 0.17 (0.001, 3.42)	Trial ended early due to “organizational issues”
Pierre (1992)^12^	970	IV	5	0.29 (0.21, 0.41)	Blood loss ≥1000 cc: 0.33 (0.14, 0.77)	Allocation to treatment vs. control done even-odd, by order of registration Those measuring blood loss were not blinded to treatment status
Abdel-Aleem (2010)^13^	1,950	IM	10	0.53 (0.39, 0.74)	Blood loss ≥1000 cc: 0.52 (0.13, 2.08) Therapeutic uterotonic needed: 0.39 (0.26, 0.58)	
Nordstrom (1997)^14^	1,000	IV	10	0.56 (0.46, 0.70)	Blood loss ≥1000 cc: 0.71 (0.45, 1.10) Therapeutic uterotonic needed: 0.57 (0.39, 0.82)	

From these trial results, it is certainly fair to say that evidence for clinical efficacy is better for 10 IU than for 5 IU (and that evidence for effectiveness of intramuscular administration of oxytocin 5 IU is particularly weak)*; it would not be fair to say the evidence is definitive.

## SAFETY

In addition to efficacy, an important consideration for uterotonics is *safety*, particular when used for labor augmentation. In many parts of the world, oxytocin (and sometimes other uterotonics) is commonly used for augmentation under unsafe conditions (not reliably ruling out mechanical obstruction, administering the drug intramuscularly or by intravenous bolus, failing to closely monitor the laboring woman, and not having timely access to emergency cesarean delivery). Because of dangers associated with uterotonic use during labor, oxytocin is designated as a high-alert medication.^15^ Wide use under unsafe conditions makes an important contribution to poor birth outcomes in South Asia^16^^–^^20^ and elsewhere.^21^

Carbetocin would certainly be no safer in this respect. Given its considerably longer half-life, arguably, deploying it widely in place of oxytocin could increase risk of adverse outcomes related to such inappropriate use—notably fetal asphyxia and uterine rupture.

## ACCESS

Oxytocin is an inexpensive medication; the median bulk price documented in the International Medical Products Price Guide from Management Sciences for Health is US$0.17 for a 10 IU amp^22^; the price for oxytocin that the United Nations Population Fund currently has posted on its website is US$0.28 per amp.^23^ And oxytocin is widely available and produced by many generic pharmaceutical manufacturers, although there are relatively few selling to low-and middle-income country markets that meet current global good manufacturing practices quality standards for oxytocin. For carbetocin to be a viable alternative to oxytocin, it would need to be similarly inexpensive and ubiquitous. It is reassuring to read that Ferring Pharmaceuticals, the sole supplier of carbetocin, hopes to make it “available in public-sector facilities of high-burden countries at an affordable and sustainable price,”^1^ but we're not there yet.

For carbetocin to be a viable alternative to oxytocin, it would need to be as inexpensive and ubiquitous as oxytocin.

## A QUIVER OF UTEROTONICS

Carbetocin is one of a suite of medicines that act on the myometrium and that, together, constitute an important set of tools for achieving better birth outcomes. It may be that carbetocin will eventually partially replace the use of some of the others for certain indications, in certain circumstances. But there will continue to be a need for oxytocin, misoprostol, tranexamic acid, and ergot alkyloids. Optimal strategies for how best to use this complement of drugs will vary by setting and need to take into account characteristics of the drugs, available evidence on effectiveness and safety, and the situation on the ground.

## Footnotes

* The Abdel-Aleem trial,^13^ with a total sample of 1,950, provides robust evidence of clinical benefit of 10 IU administered intramuscularly. The only 2 studies using 5 IU administered intramuscularly were De Groot^10^ and Poueschmann,^11^ both of which were very small and therefore lacking in adequate statistical power for our clinical endpoints of interest. As a result, they provide little evidence one way or the other about clinical efficacy of 5 IU administered intramuscularly.
